# Amphiphilic Cationic Macromolecules Highly Effective Against Multi-Drug Resistant Gram-Positive Bacteria and Fungi With No Detectable Resistance

**DOI:** 10.3389/fbioe.2020.00055

**Published:** 2020-02-14

**Authors:** Sudip Mukherjee, Swagatam Barman, Riya Mukherjee, Jayanta Haldar

**Affiliations:** Antimicrobial Research Laboratory, New Chemistry Unit and School of Advanced Materials, Jawaharlal Nehru Centre for Advanced Scientific Research, Bengaluru, India

**Keywords:** antimicrobial resistance, AMP mimics, macromolecular antimicrobial agent, polymicrobial biofilm, metabolically inactive bacteria

## Abstract

The ever increasing threats of Gram-positive superbugs such as methicillin-resistant *Staphylococcus aureus* (MRSA), vancomycin-resistant *Staphylococcus aureus* (VRSA), and vancomycin-resistant *Enterococccus faecium* (VRE) are serious matter of concern worldwide toward public health. Such pathogens cause repeated recurrence of infections through the formation of biofilms which consist of metabolically inactive or slow growing dormant bacterial population in vast majority. Concurrently, dispersal of biofilms originates even more virulent dispersed cells responsible for pathogenesis. Along with this, fungal infections most commonly associated with *Candida albicans* also created a major complicacy in human healthcare. Moreover, concomitant survival of *C. albicans* and MRSA in a multispecies biofilms created extremely complicated polymicrobial infections. Surprisingly, infections associated with single species biofilm as well as multiple species biofilm (co-existence of MRSA and *C. albicans*) are almost untreatable with conventional antibiotics. Therefore, the situation demands an urgent development of antimicrobial agent which would tackle persistent infections associated with bacteria, fungi and their biofilms. Toward this goal, herein we developed a new class of branched polyethyleneimine based amphiphilic cationic macromolecules (ACMs) bearing normal alkyl, alkyl ester and alkyl amide moieties. An optimized compound with dual activity against drug-resistant bacteria (MIC = 2–4 μg/mL) and fungi (MIC = 4–8 μg/mL) was identified with minimal toxicity toward human erythrocytes (HC_50_ = 270 μg/mL). The lead compound, ACM-A_Hex_ (**12**) displayed rapid bactericidal and fungicidal kinetics (>5 log CFU/mL reduction within 1–4 h). It also killed metabolically dormant stationary (MRSA and VRE) and persister (*S. aureus*) cells. Moreover, this compound was able to disrupt the preformed biofilm of MRSA and reduced the bacterial burden related to the dispersed cells. It showed significant proficiencies to eliminate polymicrobial biofilms of MRSA and *C. albicans*. Bacteria also could not develop any resistant against this class of membrane active molecules even after 15 days of successive passages. Taken together this class of macromolecule can be developed further as a dual therapeutic agent to combat infections associated with bacterial and fungal co-existence.

## Graphical Abstract

Advanced weapon to tackle Polymicrobial biofilm.

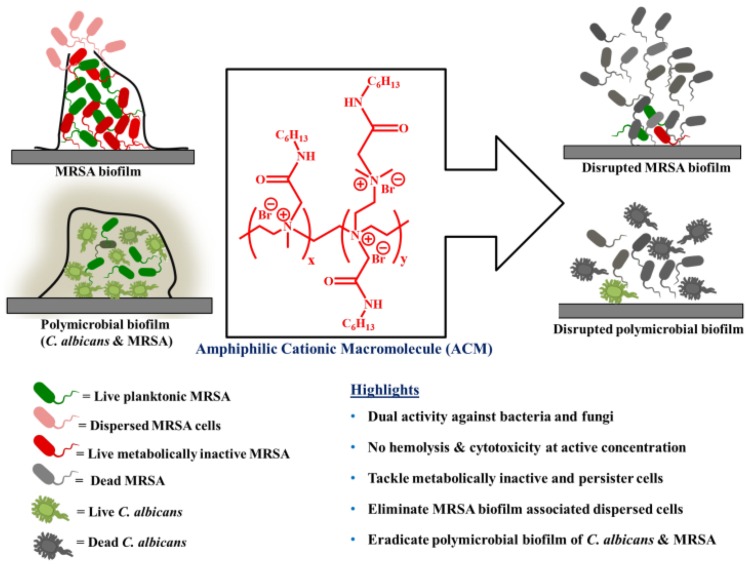

## Introduction

The overgrowing population of multidrug-resistant (MDR) pathogens has created a serious clinical ultimatum which leads the public health toward a pre-antibiotic era (Bush et al., 2011). In addition to the emergence of Gram-negative bacteria, infections caused by MDR Gram-positive bacteria have also become one of the major hurdles to tackle with conventional antibiotic therapy (Davies and Davies, 2010). In a recent report, the World Health Organization (WHO) identified a list of Gram-positive bacteria of high priority which included MRSA, VRSA, and VRE which are responsible for majority of infections in clinical settings (Willyard, 2017). The root cause of such deadly infections is the formation of bacterial biofilms, a rigid and multilayer bacterial assembly (Hall-Stoodley et al., 2004; Campoccia et al., 2013). Typically, this assembly is composed of extracellular matrix (a hurdle for antibiotics penetration) and lion’s share of it contains metabolically inactive dormant bacterial populations (Stewart and Costerton, 2001). Owing to these facts, antibiotics are inefficient to tackle chronic infections related to biofilm formation (Davies, 2003). Additionally, dispersal process in biofilms sparks more virulent dispersed cells which create a new infection foci within the host (Chua et al., 2014). Such dispersed cells are also difficult to kill with the conventional antibiotic therapy as they are distinct from the planktonic bacterial cells.

Contemporarily, fungal infection is another major problem to the human healthcare (Wilson et al., 2002). Particularly, *Candida albicans* is one of the common fungi which cause invasive infections (Brown et al., 2012). In addition to this, *C. albicans* is known to co-exist with MRSA resulting the formation of polymicrobial biofilms (Harriott and Noverr, 2009, 2011; Lohse et al., 2018). At the current situation there are limited treatment options for infection associated with such multi-species assemblies. Therefore, the situation demands an urgent need for the development of new class of antimicrobial agents with dual potency to counter bacterial as well as fungal infections associated with single and multispecies biofilm formation.

In past few decades, naturally occurring antimicrobial peptides (AMPs: a short cationic peptide) have emerged as potent, broad spectrum antimicrobials, which act as a frontline defense against a wide range of microbes (Zasloff, 2002). AMPs selectively target the negatively charged microbial membrane over zwitterionic mammalian membrane primarily through electrostatic and hydrophobic interaction (Hancock and Sahl, 2006). Owing to their membrane acting nature, microbial species find difficulties to develop resistance propensity against AMPs. However, the translation of AMPs to the clinical settings is limited majorly due to synthetic complexities, high manufacture cost and lack of *in vivo* stability. In order to address these limitations associated with AMPs, efforts have already been taken to develop several AMP-mimicking synthetic antimicrobial polymers in addition to the small molecular peptidomimetics (Ghosh et al., 2014; Konai et al., 2014). They include polynorbornene (Ilker et al., 2004), polymethacrylates (Kuroda and DeGrado, 2005), polycarbonates (Nederberg et al., 2011; Chin et al., 2018), poly-β-lactam (Porter et al., 2000; Liu et al., 2014), polymalemide (Uppu et al., 2013; Barman et al., 2019), polyamide (Yavvari et al., 2017) and many other polymeric amphiphiles (Krumm et al., 2014; Zhang et al., 2014; Konai et al., 2018; Palermo et al., 2019). The antibacterial activity is well documented for different polymeric designs, however, antifungal activity is reported for very few cases (Li et al., 2012; Liu et al., 2015; Qian et al., 2018). Typically, developing a selective antifungal agent is challenging as both the fungal and mammalian cells resemble many similarities. Hence, identifying less cytotoxic antimicrobial polymers with significant antibacterial and antifungal activity is highly desirable. Beside these hurdles, one of the major challenges is to achieve an antimicrobial polymer with ability to retain activity in complex medium such as blood plasma.

Toward this goal, herein, we report a new class of water soluble AMP-mimicking macromolecules consisting of small molecular weight (Mn ∼ 600 Da) of branched polyethyleneimine (PEI) backbone through a two-step post-functionalization strategy. It has been reported that polyethyleneimine (PEI) derivatives of higher molecular weight are potent antimicrobial agents (Hoque et al., 2015, 2019), but toxicity limits their scope for further development. Therefore, we have used small molecular weight branched PEI as a backbone polymer, aiming to obtain a lead antimicrobial agent with minimum toxicity toward mammalian cells. The molecular weight (M_n_) of the final macromolecules was bound to be in the range of 3.5–6 kDa which is much below the threshold for renal clearance (<50 kDa). In order to achieve an optimum balance of amphiphilicity (hydrophilic/hydrophobic balance), hydrophobicity was varied using various alkyl chain lengths with and without ester and amide groups. A permanent cationic charge (contributed by quaternary ammonium moiety) has been introduced through quaternization of tertiary nitrogen centers with different alkyl chain. This cationic charge is introduced for a selective interaction with the negatively charged microbial membrane over mammalian membrane. Antimicrobial activity against various drug-sensitive, drug-resistant Gram-positive bacteria and fungi was tested. Additionally, toxicity against both human red blood cell (hRBC) and HEK-293 cell were evaluated. Time kill kinetics of the optimized macromolecule was investigated against both Gram-positive bacteria and fungi. Furthermore, efficacy of the lead macromolecule was investigated against difficult-to-treat metabolically inactive different Gram-positive bacteria. The membrane active mechanism of action was also investigated against these metabolically distinct cells of bacteria and fungi. Next, biofilm disruption ability and killing efficiency against biofilm associated dispersed cells were studied. Additionally, potency of the lead molecule to eradicate polymicrobial biofilm of MRSA and *C. albicans* was investigated. To the end propensity of resistance development was also studied against MRSA superbugs.

## Materials and Methods

### Reagents and Microbial Strains

1-Butanol, 1-Hexanol, 1-Octanol, 1-Aminobutane 1-Aminohexane, 1-Aminooctane, Bromoacetyl bromide, Branched polyethyleneimine M_n_ ∼600 Da, were purchased from Sigma-Aldrich and used as received. 1-Bromobutane, 1-Bromohexane, 1-Bromooctane and 1-Bromodecane, Phosphorous pentaoxide (P_2_O_5_), potassium carbonate (K_2_CO_3_), anhydrous sodium sulfate (Na_2_SO_4_), potassium hydroxide (KOH), dichloromethane (DCM), chloroform and anhydrous diethyl ether were purchased from Spectrochem, India and were of analytical grade. DCM and chloroform were dried over P_2_O_5_ and stored over molecular sieves (4 Å). An attenuated total reflectance Fourier transform infrared (ATR FT-IR) spectrometer was used to record IR spectra using diamond as ATR crystal. Bruker (AMX-400) (400 MHz for ^1^H-NMR) spectrometer was used to record nuclear magnetic resonance (NMR) spectra in deuterated solvents. Tecan infinite pro series M200 microplate reader was used to record optical density (OD) and fluorescence intensity. *Staphylococcus aureus* and *Escherichia coli* (MTCC737 and 443) were purchased from MTCC (Chandigarh, India). Methicillin-resistant *Staphylococcus aureus* MRSA (ATCC33591), *E. faecium* (ATCC19634) and *C. albicans* (ATCC10231) were obtained from ATCC (Rockville, MD, United States). Growth media and agar for bacteria and fungal culture were provided by HIMEDIA, India. MRSA R3545, MRSA R3889, MRSA R3890 were obtained from National Institute of Mental Health and Neurosciences, Bangalore, India. VRSA-1, VRSA-4, VRE903, and VRE909 were obtained from Dr. Siddharth Chopra, CSIR-Central Drug Research Institute, Lucknow, India. Fungal strains (*C. albicans* AB226 and *C. albicans* AB399) were obtained from Anthem Biosciences, Bangalore, India.

### Synthesis

#### Synthesis of *N*-Methylated PEI (**1**)

**SCHEME 1:**
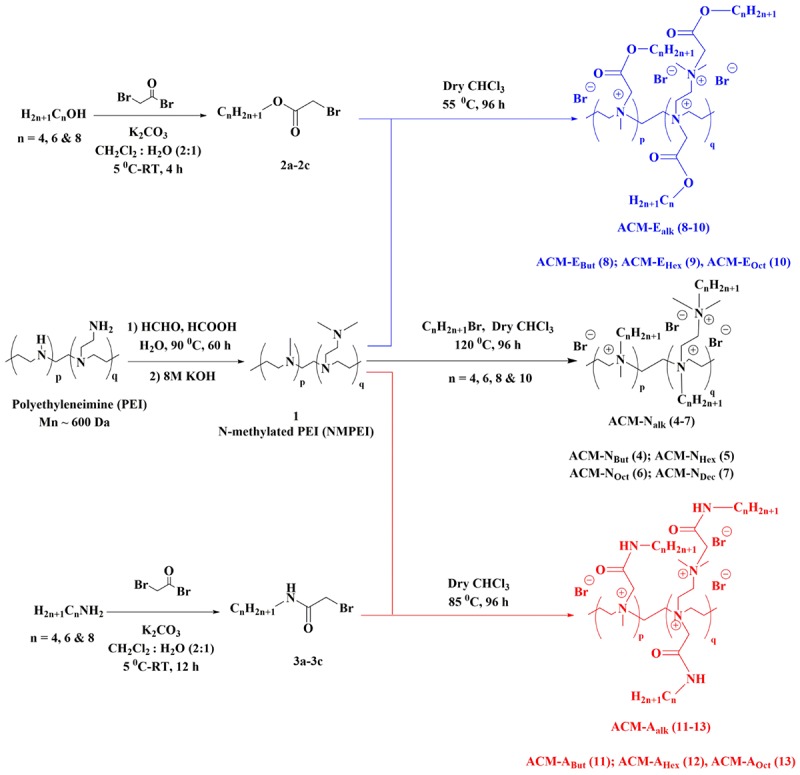
General synthesis scheme for the amphiphilic cationic macromolecules (ACMs).

Briefly, branch polyethyleneimine (PEI, 600Da) (5 g, 116 mmol) was dissolved with 20 mL of water. Then, Formic acid (17.5 mL, 464 mmol) and formaldehyde (28 mL, 348 mmol) was added to the aqueous solution of PEI (Hoque et al., 2019, p. 57). Afterward, the entire reaction mixture was refluxed at 90°C for 60 h with constant stirring. Next, the mixture was cooled down to room temperature and the pH of the solution was adjusted to 11 using aqueous solution of 8M KOH. Finally, the deprotonated *N*-methylated PEI was extracted 5 times using chloroform. To the end, the extracted organic layer was removed using Rota-evaporator to yield a brown viscous product with quantitative yield (Scheme 1). The product was characterized by FT-IR and ^1^H NMR spectroscopy (data provided in Supplementary Material).

#### General Synthetic Procedure for the Intermediates **(2a–2c)** and **(3a–3c)**

1-alkanols (5 g) or 1-aminoalkanes (5 g) were dissolved in DCM (50 mL) and 50 mL aqueous solution of K_2_CO_3_ (12.2 g, 88.3 mmol) was added to it. The binary mixture was then cooled to 4-5°C. Next, bromoacetyl bromide (7.7 mL, 88.3 mmol) was dissolved in DCM (50 mL) and was added to the mixture drop wise for 30 min. The entire biphasic reaction mixture was then stirred vigorously at room temperature for 4 and 12 h for alcohol and amines respectively. Afterward, DCM layer was separated using a separating funnel and was passed through anhydrous Na_2_SO_4_. Then the DCM solution was evaporated using rota-evaporator to obtain colorless liquids with 95–100% yield (Scheme 1). These activated alky ester or amide bromide derivatives were characterized by ^1^H-NMR and data are provided in Supplementary Material.

#### General Synthetic Procedure for ACM-N_alk_ **(4–7)**

To a solution of *N***-**methylated PEI (**1**) (17.8 mmol) in dry chloroform, 1-bromoalkanes (71.4 mmol) were added and stirred at 120°C for 96 h in a screw-top pressure tube. At the end, the excess solvent was evaporated to reduce the volume of reaction mixture. Finally, the product was precipitated by using excess anhydrous diethyl ether and the residue was washed repeatedly with the same to obtain the cationic macromolecules with 80–85% yield (Scheme 1). The final compounds were characterized by ^1^H-NMR spectroscopy (Supplementary Material).

#### General Synthetic Procedure for ACM-E_alk_ (8–10) and ACM-A_alk_ (11–13)

The synthesized activated esters and amides (**2a-2c** and **3a-3c**) (3 equivalent) were individually reacted with *N*-methylated PEI (**1**) in dry CHCl_3_ at 55–85°C in a screw-top pressure tube. At the end of 96 h, the reaction mixture was evaporated to reduce the volume. Finally, the product was precipitated by using anhydrous diethyl ether and the residue was washed repeatedly with the same to obtain the cationic macromolecules with 75–80% yield (Scheme 1). All the final compounds were characterized by ^1^H-NMR spectroscopy (Supplementary Material).

#### Antibacterial Activity

Antibacterial assay was performed by our following previously reported protocol (Konai and Haldar, 2017). Three microliter of the bacterial solution from frozen stock (–80°C) in glycerol was grown for 24 h by incubating at 37°C on NB agar plate and MacConkey agar plate for Gram-positive and Gram-negative bacteria respectively. A single bacterial colony was then allowed to grow in 3 mL nutrient broth medium for 6 h to grow mid-log phase bacteria at 37°C before starting the experiments. The mid-log phase culture was titered to give ∼10^8^ CFU/mL by the drop plating method. This culture was then diluted to ∼10^5^ CFU/mL in Muller Hinton Broth (MHB) medium. As the compounds (**4–6**, **8–9**, **11–12**) were highly soluble in water and the stock solutions were prepared in Millipore water. Whereas stock solutions of the compounds **7**, **10,** and **13** were prepared in 50% DMSO in water as they were partially water soluble. Then, all compounds were serially diluted by twofold in 96 well plates in Millipore water. After that, 180 μL of diluted bacterial suspension was added to the 96 well plate containing 20 μL of compound solution. The plates were then allowed to incubate for 24 h at 37°C. The OD was recorded at 600 nm using TECAN (Infinite series, M200 pro) plate reader. Each concentration was maintained to have triplicate values and the entire experiment was performed twice. The final minimum inhibitory concentration (MIC) value was determined by visual turbidity.

#### Antifungal Activity

Following our standard lab protocol (Ghosh et al., 2017) fungal strains were grown on YPD agar plates streaked from frozen stock (–80°C) supplemented with glycerol and incubated for 28°C for 24 h (Ghosh et al., 2014). A single fungal colony was then allowed to grow in 3 mL YPD medium for 10 h at 28°C to grow mid-log phase fungi which was titered and fungal concentration was ∼10^8^ CFU/mL. This mid-log phase culture was then diluted to ∼10^5^ CFU/mL in RPMI medium. Further procedure was similar to antibacterial assay. The O.D. was recorded after 48 h of incubation at 30°C at 600 nm using TECAN (Infinite series, M200 pro) plate reader. Each concentration of the compound was tested in triplicate and the entire experiment was performed twice. The final minimum inhibitory concentration (MIC) value was determined by visual turbidity.

#### Hemolytic Activity

According to our published protocol (Ghosh et al., 2014), briefly the macromolecules (**4–13**) were serially diluted in Millipore water in 96-well plates. Fifty microliter of 1 × PBS and 50 μL of 0.1% Triton X-100 solution were taken as negative and positive control respectively. Then human blood was collected from a healthy donor in a 10 mL heparinized tube. Then the freshly collected erythrocytes were centrifuged down and suspended in 1 × PBS (pH = 7.4). Next, 150 μL of this suspension (5 vol%) was added to the plates containing serially diluted macromolecules. These plates were then incubated at 37°C for 1 h. After that, they were centrifuged at 3500 rpm for 5 min, and 100 μL of the supernatant was then transferred to another 96-well plate to measure the absorbance at 540 nm. To calculate the hemolysis percentage, the following formula was used: (A_tret_ –A_nontret_)/(A_TX__–__tret_– A_nontret_) × 100, where A_tret_ is the absorbance of the compound-treated well, A_nontret_ the absorbance of the negative controls (without compound), and A_TX__–__tret_ the absorbance of the Triton X-100-containing well. Each concentration had triplicate values and the entire experiment was performed twice. The HC_50_ value was determined by taking the average of triplicate O.D. values and error bars represent the standard deviation.

#### Cytotoxicity

*Alamar Blue Assay:* Cytotoxicity of the macromolecules ACM-N_Dec_ (**7**) and ACM-A_Hex_ (**12**) was examined against HEK-293 cell line by Alamer Blue assay. Briefly, cells (∼10^4^ cells/well) were seeded onto the wells of a 96-well plate in DMEM media supplemented with 10% fetal bovine serum and 5% penicillin-streptomycin. Then 100 μL of serially diluted compound solution in DMEM media was added to the each well of the plates containing the cells. Same volume of media (untreated cells) and the cells treated with 0.1% (v/v) Triton-X solution was taken as positive and negative control respectively. The plates were then kept for incubation at 37°C for 24 h maintaining 5% CO_2_ atmosphere. Afterward, 10 μL of 10× Alamar Blue solution was added to each well followed by 4 h of further incubation at the same condition. Then, the absorbance was recorded at 570 nm wavelength and 600 nm wavelength was used as the reference. The percentage of cell viability was calculated using the following equation: cell viability (%) = (A_c_ – A_t_)/(A_0_ – A_t_) × 100, where A_c_ indicates the absorbance for cells treated with compound, A_t_ is the absorbance for the cells treated with 0.1% (v/v) Triton-X and A_0_ is the absorbance of the untreated cells, all at 570 nm. Each concentration had triplicate values and the entire experiment was performed twice. The average of triplicate absorbance values was plotted against concentration followed by fitting with a sigmoidal plot and the standard deviation was represented by error bars. From the curve the values were determined corresponding to 50% cell viability.

*Fluorescence microscopy:* Briefly, HEK-293 cells were seeded into the wells of 96 well tissue culture plates (∼10^4^ cells/well). Afterward, the cells were treated with ACM-A_Hex_ (**12**) at a concentration of 16 μg/mL. 0.1 vol% of Triton-X and untreated cells were taken as positive and negative control. The cells were then washed with 1× PBS for a single time and the treated and untreated cells were stained with calcein-AM (2 μM) and propidium iodide (4.5 μM) by incubating for 15 min. at 37°C under 5% CO_2_ atmosphere. After that, the cells were washed with 1× PBS to remove the excess dye and images were captured by Leica DM2500 microscope at 40× objective. To capture the images, for calcein-AM band pass filter of 500–550 nm wavelength and for propidium iodide long-pass filter of 590–800 nm wavelength was used.

#### Antibacterial Activity in Physiological Fluids

The antibacterial activity of ACM-A_Hex_ (**12**) against MRSA ATCC33591 was examined after incubating it with 50% plasma and 50% mice liver homogenate. Briefly, the compound was mixed with human blood plasma and mice liver homogenate in 1:1 ratio then incubated for different time interval (3, 6, 12, and 24 h) at 37°C. Then, these mixtures were serially twofold diluted in 1 × PBS. After that, 180 μL of diluted bacterial suspension was added to the 96 well plate containing serially diluted 20 μL of compound and plasma or liver homogenate mixture solution and incubated at 37°C for 24 h. Next, the MIC was determined similarly like the previous protocol. Each concentration had triplicate values and the experiment was performed twice. The average data was reported.

#### Bactericidal Kinetics Against Planktonic Bacteria

Time kill kinetics was performed to evaluate the bactericidal nature of the lead molecule ACM-A_Hex_ (**12**) by our standard lab protocol (Barman et al., 2019). The 6 h grown (mid-log phase) bacterial culture was diluted to ∼10^5^ CFU/mL in MHB. One hundred and eighty microliter of diluted bacterial suspensions were then incubated with 20 μL of compound solution at different concentration (8, 16, and 32 μg/mL) of the optimized compound at 37°C. Twenty microliter of Millipore water without any compound was used as negative control. Twenty microliter aliquot was taken after different time interval of 0, 1, 2, 4, and 6 h and serially 10-fold diluted in 0.9% saline. From these diluted solutions 20 μL was drop casted on nutrient agar plates and was allowed to incubate for 18 h at 37°C. The viable bacterial colonies were counted and the result plotted as Log CFU/mL vs. time (hours). The experiment was performed twice and the average data was reported. Error bars indicate standard deviation.

#### Bactericidal Kinetics Against Metabolically Inactive Bacteria

Time kill against stationary phase bacteria was determined according to our standard lab protocol (Konai et al., 2015; Konai and Haldar, 2017). To grow the stationary phase bacteria, 5 μL of mid-log phase planktonic bacteria was inoculated in 5 mL of nutrient broth (BHI for *E. faecium* and VRE) and incubated for 16 h at 37°C. Next, the stationary phase bacteria were diluted in 1 × PBS. One hundred and eighty microliter of diluted bacterial suspensions were then incubated with 20 μL of compound ACM-A_Hex_ (**12**) solution, vancomycin and linezolid at different concentration and allowed to incubate at 37°C for different time intervals. Twenty microliter of Millipore water without any compound was used as negative control. Afterward, similar procedure was performed like planktonic phase and the result was plotted as Log CFU/mL vs. time (hours). The experiment was performed twice and the average data was reported. Error bars indicate standard deviation.

#### Kinetics of Fungal Killing

Time-kill kinetics against fungi was similar to the bactericidal killing kinetics. Briefly, the 10 h grown mid-log phase culture was diluted to ∼10^5^ CFU/mL in RPMI media. Then 180 μL these diluted fungal suspensions were added to the wells of 96 well plates containing 20 μL of the compound ACM-A_Hex_ (**12**) solutions at different concentrations and incubated at 30°C for different time intervals. Similarly, then 20 μL aliquot was taken and serially 10-fold diluted in 0.9% saline. From these diluted solutions 20 μL was drop casted on YPD agar plates and was allowed to incubate for 48 h at 30°C. The viable fungal colonies were counted and the result plotted as Log CFU/mL vs. time (hours). The experiment was performed twice and the average data was reported. Error bars indicate standard deviation.

#### Anti-biofilm Activity: Biofilm Disruption, Inactivation of Dispersed Cells From Biofilm and Elimination of Metabolically Inactive Cells Within Biofilm

Biofilms were grown on sterile glass coverslips by following our earlier published protocol (Konai and Haldar, 2017). MRSA ATCC33591 and MRSA R3545 were grown in nutrient broth for 6 h to obtain mid-log phase bacteria (∼10^8^ CFU/mL). Then they were diluted in nutrient broth, supplemented with 1% glucose and 1% NaCl, to 10^5^ CFU/mL. Then 2 mL of this bacterial suspension was added to the each wells of 6 well plate containing 18 mm sterile glass coverslip. The plates were incubated at static condition for 24 h at 37°C. Biofilm containing coverslips were carefully washed with 0.9% saline and transferred to the wells of 2 mL of biofilm media containing compound ACM-A_Hex_ (**12**) at different concentration in a new 6 well plate and incubated at static condition for 24 h at 37°C. Two milliliter of the same medium was used as a untreated control and vancomycin (32 μg/mL) was taken as an antibiotic control. Afterward, these coverslips were carefully washed with 0.9% saline and kept in a new 6 well plate and the anti-biofilm activity of the compound was performed by following methods:

*CV staining*: For the biofilm biomass quantification, preformed biofilm containing coverslips were carefully washed with 0.9% saline and dried for 10–15 min. After that, they were stained with 2 mL of 0.1% crystal violet solution and incubated for 10 min. These stained biofilms were again washed with 0.9% saline and then cautiously scratched with 2 mL of 95% aqueous ethanol solution and the O.D. was recorded at 522 nm by using plate reader. The experiment was performed twice and the average data was reported. Error bars indicate standard deviation.

*Biofilm cell viability*: The biofilms were trypsinized using 2 mL of trypsin-EDTA solution in saline (1:4) and incubated for 15 min. Then the coverslips were carefully scratched and the cell suspension was then serially 10-fold diluted in saline and 20 μL of the diluted solutions was spot plated on nutrient agar plate and allowed to incubate for 18 h at 37°C. Finally, viable bacterial colonies were counted. The experiment was performed twice and the average data was reported. Error bars indicate standard deviation.

*Dispersed cell viability*: To quantify the viability of dispersed bacterial cells, 20 μl of dispersed cell suspension present in the biofilm growing media was serially 10-fold diluted in 0.9% saline and 20 μL of the diluted solutions was spot plated on nutrient agar plate and allowed to incubate for 18 h at 37°C. Afterward the viable bacterial colonies were counted. The experiment was performed twice and the average data was reported. Error bars indicate standard deviation.

*Fluorescence Microscopy*: The untreated and treated [with ACM-A_Hex_ (**12**) and vancomycin] preformed biofilm containing coverslips were washed in saline and kept in a glass slide. Next the biofilms were stained by adding 5 μL of SYTO-9 (60 μM) and PI (15 μM) mixture. Then the images were captured by Leica DM2500 microscope at 40× objective.

#### Activity Against Polymicrobial Biofilm

Activity against polymicrobial biofilm (Harriott and Noverr, 2009) was performed against *C. albicans* and MRSA. Briefly, the individual culture of mid-log phase fungi (*C. albicans* AB226) and bacteria (MRSA ATCC33591) was diluted to ∼10^6^ CFU/mL and ∼10^7^ CFU/mL in BHI media. Then 2 mL of fungal solution and 200 μL of bacterial solution were added to the wells of a 6 well plate containing sterilized glass coverslip and they were incubated at 37°C for 24 h. Afterward, coverslips were washed in saline and treated with the same volume of compound ACM-A_Hex_ (**12**) solution at different concentrations and allowed to incubate at 37°C for 24 h. The biofilms were then trypsinized using 2 mL of trypsin-EDTA solution in saline (1:10) and incubated for 15 min. Next these coverslips were carefully scratched and the cell suspension was then serially 10-fold diluted in saline and 20 μL of the diluted solutions was spot plated on amphotericin B (100 μg/mL) containing nutrient agar plate for MRSA and vancomycin (150 μg/mL) containing YPD agar plates for *C. albicans.* These plates were then incubated for 24 h at 37°C for bacteria and 48 h at 30°C for fungi. The experiment was performed twice and the average data was reported. Error bars indicate the standard deviation.

#### Membrane Active Mechanism of Action

Briefly, the planktonic and stationary phase bacterial cells of MRSA ATCC33591 (∼10^8^ CFU/mL) and *C. albicans* (∼10^8^ CFU/mL) cells were pelleted down by centrifuging at 3500 rpm for 5 min. The media was discarded and washed with 5 mM HEPES buffer (pH = 7.4) followed by resuspension in 1:1:1 ratio of 5 mM HEPES buffer, 5 mM glucose and 100 mM KCl solution. Next, 2 μM DiSC_3_ (**5**) (3,3′-Dipropylthiadicarbocyanine iodide) dye was added in the bacterial suspension and was incubated in dark for 30 min. Then 190 μL of this bacterial suspension was then transferred into the wells of black and clear bottom 96-well plate and the fluorescence intensity was recorded. Then, 10 μL of compound ACM-A_Hex_ (**12**) solution (8, 16, and 32 μg/mL) was added and the fluorescence intensity was recorded for 26 min. Same volume of Millipore water without any compound was taken as negative control.

#### Live Dead Assay Against Bacteria

In brief 1 mL of planktonic bacterial cells (MRSA ATCC33591) was pelleted down by centrifuging at 3500 RPM for 5 min. The media was discarded and the cells were washed with 0.9% saline followed by resuspension in 1 mL saline. Then ACM-A_Hex_ (**12**) was added to this suspension to obtain a final concentration of 16 μg/mL. Then it was allowed to incubate for 2 h at 37°C. Afterward, the solution was centrifuged and the cells were re-suspended in saline followed by the addition of SYTO-9 and PI to obtain a final concentration of 3 and 15 μM respectively. This dye containing solution was incubated in dark for 15 min. The solution was centrifuged and washed with saline to remove the excess dye. Next 5 μL of this solution was subjected to fluorescence microscopy and the images were captured by Leica DM2500 microscope at 100× objective. For SYTO-9 band pass filter of 450–490 nm wavelength and for propidium iodide filter of 515–560 nm wavelength was used.

#### Live Dead Assay Against Fungi

This experiment was performed similar as Live Dead Assay against Bacteria. In brief 1 mL of fungal cells (*C. albicans* ATCC10231) was pelleted down by centrifuging at 3500 RPM for 5 min. The media was discarded and the cells were washed with 0.9% saline followed by resuspension in 1 mL saline. Then ACM-A_Hex_ (**12**) was added to this suspension to obtain a final concentration of 32 μg/mL. Then it was allowed to incubate for 4 h at 30°C. Afterward, the solution was centrifuged and the cells were re-suspended in saline followed by the addition of SYTO-9 and PI to obtain a final concentration of 3 and 15 μM respectively. This dye containing solution was incubated in dark for 30 min. The solution was centrifuged and washed with saline to remove the excess dye. Next 5 μL of this solution was subjected to fluorescence microscopy and the images were captured by Leica DM2500 microscope at 40× objective. For SYTO-9 band pass filter of 450–490 nm wavelength and for propidium iodide filter of 515–560 nm wavelength was used.

#### Development of Resistance Propensity

Resistance development study was performed by our previously published protocol (Barman et al., 2019). In brief, the MIC of ACM-A_Hex_ (**12**) and norfloxacin was evaluated by the protocol mentioned for antibacterial assay. In the subsequent days bacterial solution from the sub-MIC concentration was diluted to ∼10^5^ CFU/mL and was used for another MIC determination. After 15 days of serial passages the increment in MIC was plotted vs. the number of days. Here each concentration was tested in triplicate and fold of increase in MIC was obtained by dividing first MIC value from MIC value of every subsequent day.

#### Statistical Analysis

Statistical analysis was performed by using Graph Pad Prism version 8.3.0 software. The reported values were expressed as average ± standard deviation. The *p* < 0.05 was considered to be statistically significant.

## Results

### Design and Synthesis

The synthesis of amphiphilic cationic macromolecules (ACMs) was carried out through simple three synthetic steps involving post-functionalization strategy as shown in Scheme 1. At first *N*-methylated polyethleneimine (NMPEI) (**1**) was synthesized by Eschweiler-Clarke methylation of the precursor branched polyethyleneimine (PEI). This process led to the conversion of all primary and secondary amines moieties of PEI to tertiary amines. One new peak at 2.2 ppm (corresponding to the –N(C*H*_3_)– group) and another broad peak in the region of 2.3–2.5 ppm (correspond to –N(*CH_2_CH*_2_)– groups) have appeared in ^1^H-NMR spectrum of *N*-methyl polyethyleneimine (NMPEI) (**1**). Further, in FT-IR spectrum, peaks at 3280 and 1600 cm^–1^ corresponding to the N–H stretching and bending of parent PEI respectively was completely disappeared. The appearance of new peaks and disappearance of existing peaks in ^1^H-NMR and FT-IR spectrum respectively therefore confirmed the full conversion of primary and secondary amines to tertiary amines in NMPEI (**1**) intermediate. Afterward, NMPEI (**1**) was reacted with different normal alkyl bromides (1-Butyl bromide, 1-Hexyl bromide, 1-Octyl bromide and 1-Decyl bromide) at 120°C for 96 h in order to produce first set of amphiphilic cationic macromolecules, ACM-N_alk_ (**4–7**). Herein, alkyl long chains were varied from butyl to decyl in order to establish the effect of hydrophobicity on antimicrobial activity and toxicity.

Next, to find out the effect of ester and amide moiety in the pendant alkyl chain toward their antimicrobial activity and toxicity we also have synthesized another two sets of amphiphilic cationic macromolecules consisting ester moieties, ACM-E_alk_ (**8–10**) and the amide moieties, ACM-A_alk_ (**11–13**). Initially, different alcohols (1-Butanol, 1-Hexanol, and 1-Octanol) and amines (1-Butanamine, 1-Hexanamine and 1-Octanamine) were reacted with bromoacetyl bromide to synthesize the activated esters (**2a–2c**) and activated amides (**3a–3c**). Subsequently, these activated esters and amides were reacted with NMPEI (**1**) to synthesize other sets of cationic amphiphilic macromolecules ACM-E_alk_ (**8–10)** and ACM-A_alk_ (**11–13**). In this case also similar long chain variation from butyl to octyl was executed. All the final compounds were characterized through ^1^H-NMR and FI-IR spectroscopy. The disappearance of peak in the region of 2.3–2.5 ppm corresponding to –N(*CH_2_CH_2_*)– group of the NMPEI confirmed the complete quaternization in all final macromolecules. This result therefore suggested the 100% degree of quaternization (DQ) of all the final macromolecules (**4–13**). The molecular weight (M_n_) of the macromolecules was determined through ^1^H-NMR spectroscopy and was found to be in the range of 3.5–6 kDa (Supplementary Table S1 and Supplementary Material). Most of the amphiphilic cationic macromolecules (**4–6**, **8–9,** and **11–12**) were water soluble (>10 mg/mL) however the macromolecules **7**, **10,** and **13** were partially soluble in water.

### Antimicrobial Activity

Initially, antimicrobial activity of the cationic amphiphilic macromolecules, ACMs (**4–13**) was evaluated against ATCC and MTCC strains of Gram-positive bacterium (MRSA), Gram-negative bacterium (*E. coli*) and fungi (*C. albicans*) as depicted in Table 1. The antimicrobial activity was performed through broth dilution assay and was expressed in terms of minimum inhibitory concentration (MIC), the minimum concentration of the compounds required for microbial growth inhibition. Mostly all the macromolecules displayed appreciable antimicrobial activity against MRSA (MIC = 2–32 μg/mL), *E. coli* (MIC = 4–128 μg/mL) and *C. albicans* (MIC = 1–32 μg/mL). In case of first set of amphiphilic cationic macromolecules, where normal aliphatic alkyl chains with varying hydrophobicity (ACM-N_alk_: **4–7**) were introduced into the *N*-methyl PEI backbone in order to investigate the effect of hydrophobicity on antimicrobial activity. The compound consisting of shorter alkyl chain (butyl), ACM-N_But_ (**4**) did not display any antimicrobial activity even at 1024 μg/mL against MRSA, *E. coli* and *C. albicans*. While a moderate activity was observed for the compound consisting hexyl chain with the MIC values of 16 μg/mL against MRSA, 128 μg/mL against *E. coli* and 32 μg/mL against *C. albicans.* Further increment of hydrophobicity by incorporating octyl long chain in case of ACM-N_Oct_ (**6**), did not increase the activity much against MRSA (MIC = 8–16 μg/mL) and *C. albicans* (MIC = 32 μg/mL) whereas increase in activity was observed against *E. coli* (MIC = 32 μg/mL). The macromolecule, ACM-N_Dec_ (**7**) bearing higher hydrophobic decyl chain displayed good activity with the MIC value of 2 μg/mL against MRSA, 4–8 μg/mL against *E. coli* and 1 μg/mL against *C. albicans*.

**TABLE 1:** Antimicrobial and hemolytic activity of the amphiphilic cationic macromolecules (ACMs).

Compounds	Minimum inhibitory concentration, MIC (μg/mL)			HC_50_ (μg/mL)	Selectivity Index	
	MRSA	*E. coli*	*C. albicans*		HC_50_/MIC_MRSA_	HC_50_/MIC_*C. albicans*_
ACM-N_But_ **(4)**	>1024	>1024	>1024	>2048	–	–
ACM-N_Hex_ **(5)**	16	128	32	>2048	>128	>64
ACM-N_Oct_ **(6)**	8–16	32	32	>2048	>128–256	32
ACM-N_Dec_ **(7)**	2	4–8	1	260	130	260
ACM-E_But_ **(8)**	128	>1024	>1024	>2048	>16	–
ACM-E_Hex_ **(9)**	8–16	32	16	750	47–93	93
ACM-E_Oct_ **(10)**	4	8	4	25	∼6	∼6
ACM-A_But_ **(11)**	16–32	256	>1024	>2048	>64–128	–
ACM-A_Hex_ **(12)**	2–4	8	4	270	67–135	67
ACM-A_Oct_ **(13)**	4	16	4	45	∼10	2.5
Vancomycin	0.5–1	^a^N.D.	N.D.	N.D.	N.D.	N.D.
Colistin	N.D.	1	N.D.	N.D.	N.D.	N.D.
Amphotericin B	N.D.	N.D.	<0.25	N.D.	N.D.	N.D.

*^*a*^*N.D. stands for not determined.**

Next, to investigate the effect of ester and amide moieties on the antimicrobial activity, we synthesized other two sets of macromolecules consisting ester (ACM-E_alk_: **8–10**) and amide (ACM-A_alk_: **11–13**) functionalities in the pendant side chain of *N*-methyl PEI. In this case, the variation of alkyl chain hydrophobicity of ester and amide group also resulted the similar observation, i.e. in general, antimicrobial activity improves with increasing hydrophobicity of alkyl chain. The macromolecule ACM-E_But_ (**8**) bearing shorter alkyl chain (butyl ester) exhibited moderate to no activity against MRSA, *E. coli* and *C. albicans.* However, further increment of hydrophobicity by introducing hexyl ester moiety in case of ACM-E_Hex_ (**9**) 16–32-fold improved activity was observed with MIC values of 8–16, 32, and 16 μg/mL against MRSA, *E. coli* and *C. albicans* respectively. Likewise the previous analog, ACM-E_Oct_ (**10**) (consisting octyl ester functionality) showed enhanced antimicrobial activity (MIC = 4 μg/mL for MRSA, 8 μg/mL for *E. coli*, and 4 μg/mL for *C. albicans*) against all the tested pathogens. Moving from ester to amide bearing macromolecules, ACM-A_But_ (**11**) consisting of butyl amide residue displayed better antimicrobial activity against MRSA (MIC = 16–32 μg/mL) and *E. coli* (MIC = 256 μg/mL) compared to its butyl ester bearing analog ACM-E_But_ (**8**) while similar activity was observed against *C. albicans* (MIC > 1024 μg/mL). In the same way, ACM-A_Hex_ (**12**) (consisting higher hydrophobic hexyl amide moiety) displayed improved MIC values of 2–4 μg/mL against MRSA, 8 μg/mL against *E. coli* and 4 μg/mL against *C. albicans* compared to its previous butyl amide analog (ACM-A_But_). Interestingly, hexyl amide containing macromolecule (ACM-A_Hex_**: 12**) also displayed better antimicrobial efficacy compared to its same ester analog bearing macromolecule (ACM-E_Hex_). However, increase of further hydrophobicity in case of octyl amide bearing macromolecule ACM-A_Oct_ (**13**) resulted not much improvement of activity (MIC in the range of 4–16 μg/mL) against the tested pathogens. Herein, the approved antibiotics showed good activity such as vancomycin against MRSA with MIC = 0.5–1 μg/mL, colistin against *E. coli* with MIC = 1 μg/mL and amphotericin B against *C. albicans* with MIC < 0.25 μg/mL.

### Hemolytic Activity and Selectivity

**FIGURE 1:**
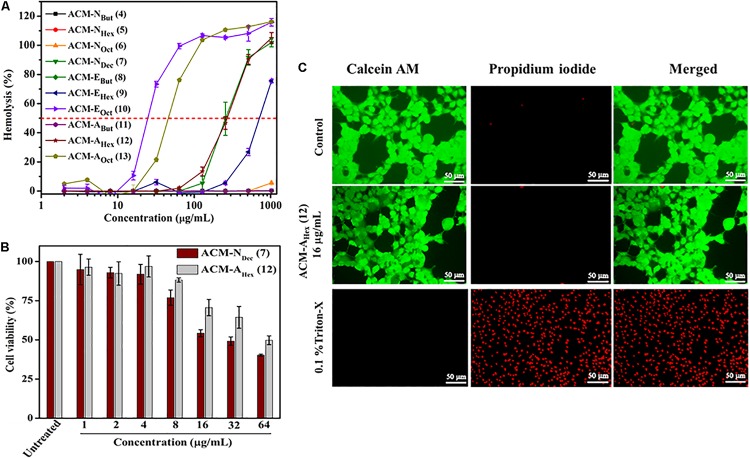
*In vitro* toxicity of amphiphilic cationic macromolecules (ACMs): **(A)** toward human erythrocytes (*p* < 0.0001, two-way ANOVA); **(B)** toward HEK-293 cells (*p* = 0.0232, two-way ANOVA). Here each value represents average of three different experimental values and error bar indicates standard deviation. **(C)** Fluorescence microscopy images of HEK-293 cells upon compound treatment. Scale bar is 50 μm.

Potent antimicrobial activity with minimal toxicity is one of the important criterions for the development of any antimicrobial agents. Initially, toxicity of this class of macromolecules was evaluated against human red blood cells (hRBCs) and was expressed in terms of HC_50_, the concentration of the compound corresponding to lysis of 50% hRBCs (Table 1 and Figure 1A). This class of macromolecules (ACMs: **4–13**) displayed overall HC_50_ values in the range of 25 to > 2048 μg/mL. In detail, most of the compounds bearing normal aliphatic alkyl moieties ACM-N_alk_ (**4–6**) displayed almost no toxicity (<5% hemolysis) even at the highest tested concentration (2048 μg/mL). Interestingly in this case, enhancement of hydrophobicity (moving from butyl to hexyl and octyl long chain consisting macromolecules) did not display significant impact toward hRBC lysis, although the antimicrobial activity was improved drastically. The compound bearing decyl chain, ACM-N_Dec_ (**7**) did not display any toxicity at 64 μg/mL and showed a minimal hRBC lysis (∼5%) at 128 μg/mL. While the HC_50_ value was found to be 260 μg/mL despite exhibiting a broad spectrum antimicrobial activity (MIC) at much lower concentration of 1–8 μg/mL. In case of macromolecules bearing alkyl ester group ACM-E_But_ (**8**), ACM-E_Hex_ (**9**), ACM-E_Oct_ (**10**) displayed HC_50_ values of >2048, 750, and 25 μg/mL respectively. However, only 5% hemolysis was observed at 256 μg/mL for the macromolecule bearing hexyl ester moiety (**9**) which displayed a considerable antimicrobial activity. Simultaneously, the molecule consisted of butyl amide functionality ACM-A_But_ (**11**) exhibited no toxicity toward hRBCs like the butyl ester containing macromolecule. The hexyl (ACM-A_Hex_; **12**) and octyl (ACM-A_Oct_; **13**) amide bearing molecules displayed HC_50_ values of 270 and 45 μg/mL respectively. Particularly, ACM-A_Hex_ (**12**) showed very minimal hemolysis (<2%) at 64 μg/mL whereas it exhibited a good antimicrobial activity at much lower concentration in the range of 2–8 μg/mL.

In order to select the lead macromolecules for further investigation of antibacterial and antifungal efficacy in detail, selectivity index (SI) (ratio of HC_50_ and MIC) was calculated considering the MIC values of macromolecules against both MRSA and *C. albicans*. Most of the normal alkyl chain bearing macromolecules (ACM-N_Alk_: **5–7**) showed moderate to good selectivity index in the range of >128–256 and 32–260 against MRSA and *C. albicans* respectively. ACM-N_Oct_ (**6**) and ACM-N_Dec_ (**7**) displayed appreciable selectivity against MRSA with a selectivity index of >128–256 and 130 respectively. Simultaneously, they also exhibited good selectivity index (SI of ACM-N_Oct_ = 32 and ACM-N_Dec_ = 260) against *C. albicans.* In general ester bearing molecules (**8–10**) showed relatively low selectivity index compared to normal alkyl chain bearing macromolecules. However, hexyl ester containing ACM-E_Hex_ (**9**) revealed relatively moderate selectivity (SI = 47–93) against both MRSA and *C. albicans*. On the contrary, amide bearing macromolecules exhibited better selectivity in comparison to the molecules consisting ester analog. Besides the butyl amide containing macromolecule (ACM-A_B__ut_: **11,** selectivity index = 64–128), a good selectivity was observed for ACM-A_Hex_ (**12**) bearing hexyl amide with a selectivity index of 67–135 against MRSA and 67 against *C. albicans*. Therefore, based on the selectivity index, we have selected two molecules (ACM-N_Oct_; **6** and ACM-N_Dec_; **7**) from ACM-N_Alk_ series, one molecule (ACM-E_Hex_; **9**) from the ACM-E_Alk_ series and one molecule (ACM-A_Hex_; **12**) from the ACM-A_Alk_ series for the further studies.

### Antimicrobial Activity Against Clinical Isolates of Drug-Resistant Gram-Positive Bacteria and Fungi

Considering the emergence and severity of MRSA, VRSA, VRE, and C. *albicans* associated infections, we therefore investigated the antimicrobial activity against their different clinical isolates for the aforementioned four selective macromolecules ACM-N_Oct_ (**6**), ACM-N_Dec_ (**7**), ACM-E_Hex_ (**9**), and ACM-A_Hex_ (**12**) (Table 2). In detail, ACM-N_Oct_ (**6**) and ACM-E_Hex_ (**9**) displayed antimicrobial activity against different clinical isolates of MRSA (MIC = 8–16 μg/mL), VRSA (MIC = 8 μg/mL), VRE (MIC = 16–64 μg/mL) and *C. albicans* (16–64 μg/mL). On the other hand, ACM-N_Dec_ (**7**) and ACM-A_Hex_ (**12**) showed better activity with MIC values of 2–4, 4–8, and 1–8 μg/mL against all the tested clinical isolates of MRSA, VRSA, VRE, and *C. albicans* respectively. In order to compare the efficacy of the macromolecules, methicillin, vancomycin, amphotericin B and fluconazole were included in our studies as a positive control. Overall these result suggested that compound consisting aliphatic decyl long chain ACM-N_Dec_ (**7**) and hexyl amide conjugated macromolecule ACM-A_Hex_ (**12**) have potent efficacy against drug-resistant Gram-positive bacteria and fungi among the four best selective compounds. Hence these two compounds ACM-N_Dec_ (**7**) and ACM-A_Hex_ (**12**) were selected for further detailed investigations.

**TABLE 2:** Antimicrobial activity of amphiphilic cationic macromolecules (ACMs) against clinical isolates of drug-resistant Gram-positive bacteria and fungi.

Microbes	Minimum inhibitory concentration, MIC (μg/mL)							
	ACM-N_Oct_ (6)	ACM-N_Dec_ (7)	ACM-E_Oct_ (9)	ACM-A_Hex_ (12)	Methicillin	Vancomycin	Amphotericin B	Fluconazole
MRSA R3545	8	2	8	4	16–32	1	^*a*^N.D.	N.D.
MRSA 3889	8	2	8	4	16–32	1	N.D.	N.D.
MRSA R3890	16	4	16	4	>32	1	N.D.	N.D.
VRSA 1	8	2	8	4	N.D.	512	N.D.	N.D.
VRSA 4	8	2	8	4	N.D.	512	N.D.	N.D.
VRE903	64	8	16	4	N.D.	> 1024	N.D.	N.D.
VRE909	64	8	16	4	N.D.	512	N.D.	N.D.
*C. albicans* AB226	32	4	32	8	N.D.	N.D.	0.25	>256
*C. albicans* AB399	64	4	64	8	N.D.	N.D.	0.25	>256

**N.D., stands for not determined.**

### Cytotoxicity

In order to have a clear idea on the toxicity profile, further cytotoxicity of two best lead macromolecules, ACM-N_Dec_ (**7**) and ACM-A_Hex_ (**12**) were examined against human embryonic kidney (HEK-293) cell line by Alamar blue assay. As a measure of toxicity, EC_50_ (concentration corresponds to 50% cell viability) values of these compounds were determined (Figure 1B). The compound with normal decyl long chain, ACM-N_Dec_ (**7**) displayed EC_50_ value of 30 μg/mL. Whereas, hexyl amide bearing macromolecule, ACM-A_Hex_ (**12**) exhibited relatively reduced cytotoxicity toward HEK-293 cells with an EC_50_ value of 60 μg/mL. Therefore, we consider the compound ACM-A_Hex_ (**12**) as the optimized lead molecule among all the amphiphilic cationic macromolecules (ACMs: **4–13**).

The toxicity of the optimum compound, ACM-A_Hex_ (**12**) was further studied through fluorescence microscopy by simultaneous staining of HEK-293 cells with calcein-AM and propidium iodide (PI). It can be clearly visualized from Figure 1C that most of the cells were alive even after compound treatment at 16 μg/mL which was 2-8 times higher than its MIC value. No cells can be seen which are labeled with red fluorescence corresponds to dead cells which suggested the minimal toxic nature of this macromolecule toward mammalian cells.

### Antibacterial Activity in Physiological Fluids

One of the major limitations of antimicrobial peptides (AMPs) is the loss of antibacterial activity in physiological fluids owing to protease degradation. The compound which sustains its antibacterial potency in such conditions would be highly desirable. Toward this goal, antibacterial activity (MIC) of the lead compound ACM-A_Hex_ (**12**) was evaluated against MRSA ATCC33591 after incubating the compound with 50% human plasma and mice liver homogenate at different time interval (3, 6, 12, and 24 h). Pre-incubation of compound in 50% plasma resulted in slight increase of MIC (two-fold) within 3–24 h time duration (Supplementary Figure S1 and Supplementary Material). When the compound was incubated in 50% liver homogenate no change in MIC was noticed upto 6 h of pre-incubation while only twofold increment of MIC was found after 12–24 h incubation. This result therefore indicated that unlike AMPs this macromolecule can be used in *in vivo* settings in order to tackle systemic infections.

### Bactericidal Kinetics Against Planktonic Bacteria

**FIGURE 2:**
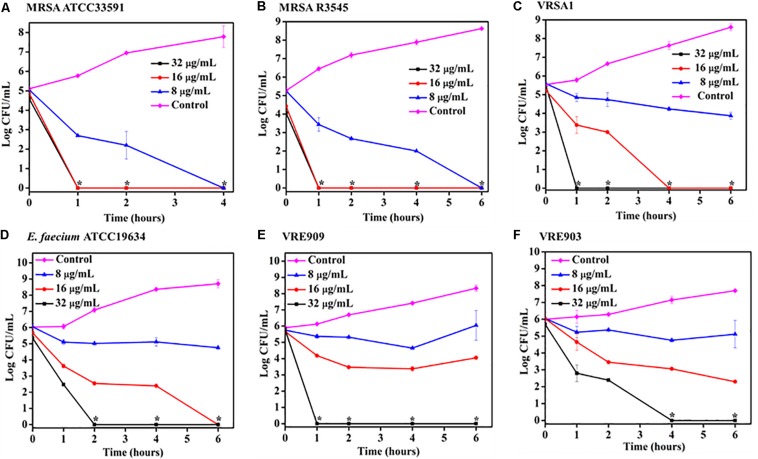
Time kill kinetics of ACM-A_Hex_ **(12)** against planktonic cells **(A)** MRSA ATCC33591; **(B)** MRSA R3545; **(C)** VRSA1; **(D)** *E. faecium* ATCC19634; **(E)** VRE909; **(F)** VRE903. (Asterisks corresponds to <50 CFU/mL). Each point represents average of four different values and error bar indicates standard deviation (*p* < 0.0001, two-way ANOVA).

Time kill kinetics of ACM-A_Hex_ (**12**) was performed against different MRSA, VRSA, and *E. faecium* (both vancomycin susceptible and resistant) strains including the clinical isolates (Figure 2). In general, the compound was rapidly bactericidal in nature. The complete killing (>5 Log CFU/mL reduction) of MRSA ATCC33591 was observed within 4 h at 8 μg/mL (2 × MIC) (Figure 2A). At the same concentration, more than 5 Log CFU/mL reduction (complete killing) was observed in case of MRSA R3545 clinical isolate within 6 h (Figure 2B). However, at 16 (4 × MIC) and 32 μg/mL (8 × MIC), the macromolecule displayed even faster bactericidal kinetics with complete killing of both the MRSA strains within 1 h. In case of, VRSA at 16 μg/mL ACM-A_Hex_ (**12**) was able to kill the bacteria completely (∼5.5 Log CFU/mL reduction) within 4 h (Figure 2C). A complete killing was observed within 1 h at a higher concentration of 32 μg/mL. At 32 μg/mL *E. faecium* was completely killed within 2 h (Figure 2D). Simultaneously, the compound was able to kill both VRE909 and VRE903 within 1 and 4 h at 32 μg/mL respectively (Figures 2E,F). Overall, these results demonstrated rapidly bactericidal nature of the lead macromolecule against multi-drug resistant Gram-positive bacteria.

### Bactericidal Kinetics Against Metabolically Inactive Bacteria

**FIGURE 3:**
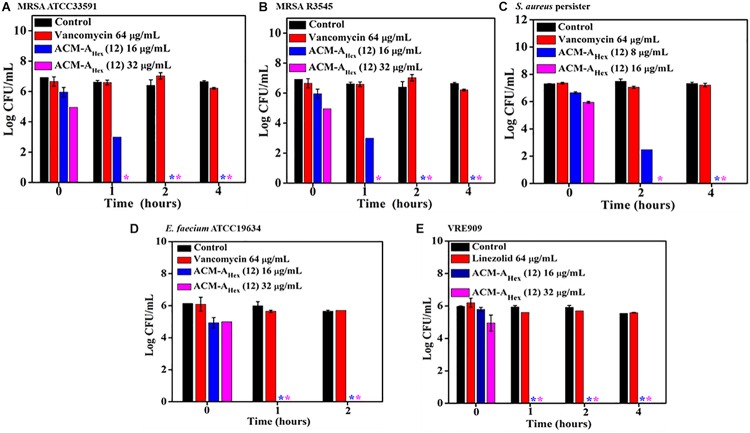
Time kill kinetics of ACM-A_Hex_ (**12**) against **(A)** stationary phase MRSA ATCC33591; **(B)** stationary phase MRSA R3545; **(C)** persisters of *S. aureus* MTCC737; **(D)** stationary phase *E. faecium* ATCC19634; **(E)** stationary phase VRE909. (Asterisks corresponds to <50 CFU/mL). Each value represents average of four different values and error bar indicates standard deviation (*p* < 0.0001, two-way ANOVA).

The emergence of metabolically inactive or dormant bacteria has created a huge complicacy in clinical settings. One such metabolically inactive species is stationary phase bacteria which slow down all biological processes. Therefore, conventional antibiotics which target various cellular processes (such as cell wall biosynthesis, protein synthesis, DNA replication etc.) become ineffective to kill such stationary cells. On the other hand, persister, another type of metabolically inactive species is a sub-population of planktonic bacteria survivors after antibiotic treatment. Hence, the situation demands an urgent development of antibacterial agents effective to kill such dormant bacteria. Toward this aim, we inspected the efficacy of our lead compound, ACM-A_Hex_ (**12**) against stationary phase MRSA, *E. faecium*, VRE, and *S. aureus* persister by performing bactericidal killing kinetics. The efficacy of the lead compound was compared with vancomycin and linezolid, last resort of Gram-positive antibiotics (Figure 3). Vancomycin even at very high concentration 64 μg/mL (64 × MIC) was completely ineffective to kill stationary and persister cells of MRSA and *S. aureus* respectively whereas it was highly active against their planktonic cells. On the contrary, ACM-A_Hex_ (**12**) at 16 μg/mL (4 × MIC) fully eradicated (∼6 Log reduction) stationary phase cells of MRSA ATCC33591 and MRSA R3545 within 1–2 h (Figures 3A,B). This also eliminated difficult-to-kill persister cells of *S. aureus* within 2 h at 16 μg/mL (4 × MIC) (Figure 3C). Similarly, stationary phase *E. faecium* ATCC19634 and VRE909 was also killed entirely at 16 μg/mL (4 × MIC) concentration of the compound (Figures 3A,B). In case of VRE909 stationary cells, linezolid was inefficient even at 64 μg/mL. Taken together, these results suggested that the macromolecule is highly efficient to tackle stationary as well as persister phase bacteria.

### Time-Kill Kinetics Against Fungi

**FIGURE 4:**
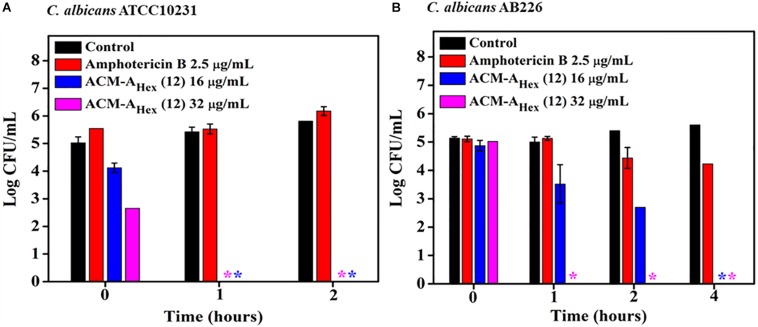
Time kill kinetics of ACM-A_Hex_ (**12**) against **(A)** *C. albicans* ATCC10231 and **(B)** *C. albicans* AB226. (Asterisks corresponds to <50 CFU/mL). Each point represents average of four different values and error bar indicates standard deviation (*p* < 0.0001, two-way ANOVA).

To evaluate the fungicidal nature of the lead compound, ACM-A_Hex_ (**12**) time-kill kinetics was performed against *C. albicans*. Likewise the rapid bactericidal nature, it also exhibited a rapid fungicidal nature. ACM-A_Hex_ (**12**) was able to kill (∼5 Log reduction) fungi completely at 32 μg/mL (8 × MIC) within 1 h against both the strains of *C. albicans* (ATCC10231 and AB226) (Figures 4A,B). In contrast, amphotericin B did not display any reduction of fungal cell viability at 2.5 μg/mL which was 10 times higher than its MIC. This result therefore demonstrated that the lead molecule may be a promising candidate to tackle both bacterial and fungal infections.

### Anti-biofilm Activity: Biofilm Disruption, Inactivation of Dispersed Cells From Biofilm and Elimination of Metabolically Inactive Cells Within Biofilm

**FIGURE 5:**
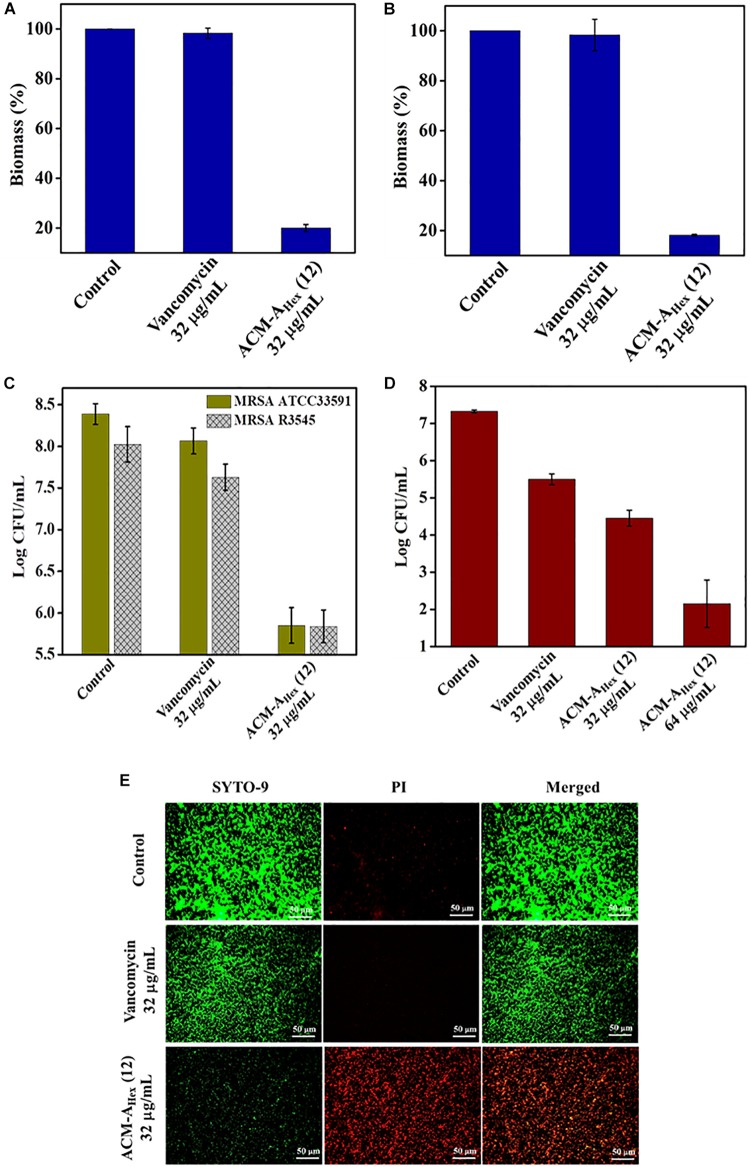
Anti-biofilm activity of ACM-A_Hex_ (**12**). Quantification of biofilm biomass after crystal violet straining for **(A)** MRSA ATCC33591 (*p* = 0.0046, one-way ANOVA) and **(B)** MRSA R3545 (*p* = 0.0007, one-way ANOVA). Quantification of bacterial cells after treatment for **(C)** MRSA ATCC33591 (*p* = 0.014, one-way ANOVA) and MRSA R3545 (*p* = 0.0198, one way ANOVA). **(D)** Activity against dispersed cells originated from MRSA ATCC33591 biofilm (*p* = 0.0114, one-way ANOVA). Herein, each value represents average of three different experimental values and error bar indicates standard deviation. **(E)** Fluorescence microscopy images of MRSA ATCC33591 biofilm by SYTO-9 and PI staining. Scale bar is 50 μm.

Bacterial biofilm formation is one of the emerging issues in clinical settings which mostly accounts for complicated and chronic infections. Biofilm is a multicellular microbial assembly composed of self-produced extracellular matrix with diffusion barriers for antimicrobial agents and predominance of metabolically inactive or dormant bacterial cells. In addition to this, dispersed cells originated upon the mature biofilm dispersal were found to be distinct in nature in comparison to the planktonic and biofilm embedded bacterial cells. Such dispersed cells are highly virulent compared to the planktonic cells (Chua et al., 2014). Due to the, aforesaid facts, biofilm associated infections are almost untreatable by conventional antibiotics. Hence, this scenario necessitates the development of anti-bacterial agents with potent anti-biofilm efficacy. Therefore, we evaluated anti-biofilm properties of the optimized macromolecule ACM-A_Hex_ (**12**) against MRSA including clinical isolates through crystal violet straining as well as by counting bacterial cell viability within the biofilm and in dispersed cells (Figure 5). The compound was found to eradicate ∼80% of biofilm biomass at 32 μg/mL for both the preformed the biofilm of MRSA ATCC33591 and MRSA R3545 strains. On the contrary, vancomycin displayed hardly any biofilm biomass reduction at the same concentration (Figures 5A,B).

Furthermore, this macromolecule was highly efficient to kill the bacteria embedded within the biofilm matrix. It displayed 2.5 Log and 3 Log reduction of bacterial burden of MRSA R3545 and MRSA ATCC33591 respectively within the biofilm (Figure 5C). More importantly, the compound was also able to eliminate virulent dispersed cells of MRSA ATCC33591 (∼3 Log reduction) while vancomycin was almost incompetent to kill them (Figure 5D).

Next the extent of biofilm disruption was visualized by fluorescence microscopy studies with simultaneous staining of SYTO-9 (green fluorescence) and PI (red fluorescence) (Figure 5E). In the untreated and the vancomycin (32 μg/mL) treated cases the biofilm showed green fluorescence, displaying the presence of live bacterial cells. In contrast, for compound treated case, a strong red fluorescence was observed, indicating the presence of dead bacterial cell associated with biofilm matrix. Collectively, these results demonstrated that the lead compound showed significant promises to be developed as future anti-biofilm therapeutics.

### Activity Against Polymicrobial Biofilm

**FIGURE 6:**
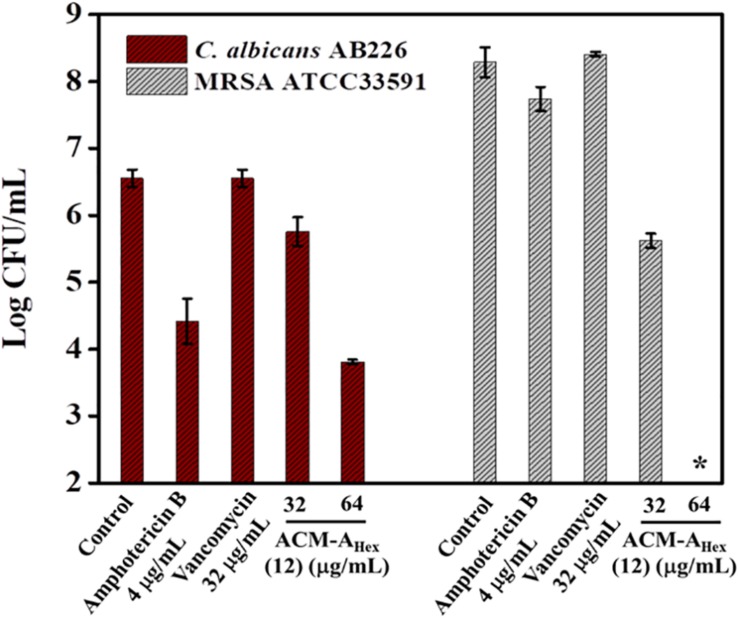
Quantification of microbial cells embedded within Polymicrobial biofilm of *C. albicans* AB226 and MRSA ATCC33591. (Asterisks corresponds to <50 CFU/mL). Each value represents average of two different experimental values and error bar indicates standard deviation (*p* < 0.0001, two-way ANOVA).

Dual existence of MRSA and *C. albicans* give rise to the formation of polymicrobial biofilms which is not fully curable even with multiple antibiotics treatment. Thus, antimicrobial agent with efficiency to tackle such multispecies infections is of high demand. Dual efficacy of our lead macromolecule ACM-A_Hex_ (**12**) against both bacteria and fungi therefore motivated us to evaluate its efficacy against polymicrobial biofilms. Upon the treatment with this compound, we could observe a significant reduction of both fungal and bacterial burden (Figure 6). In case of fungi, around 2.5 log reduction was found at 64 μg/mL of the compound whereas amphotericin B displayed comparatively less reduction (2 log) even at its high therapeutic concentration (>16 × MIC). On the other hand, the compound displayed 2 log reduction of MRSA at 32 μg/mL and completely killed MRSA (∼8 log reduction) at 64 μg/mL. Whereas vancomycin was totally inefficient even at 32 μg/mL. None of the antibiotics used in this study were able to kill simultaneously both the organisms. Interestingly, our lead molecule ACM-A_Hex_ (**12**) successfully brought down the microbial burden associated with both *C. albicans* and MRSA. This result demonstrated the unique properties of this class of macromolecule.

### Membrane Active Mechanism of Action

**FIGURE 7:**
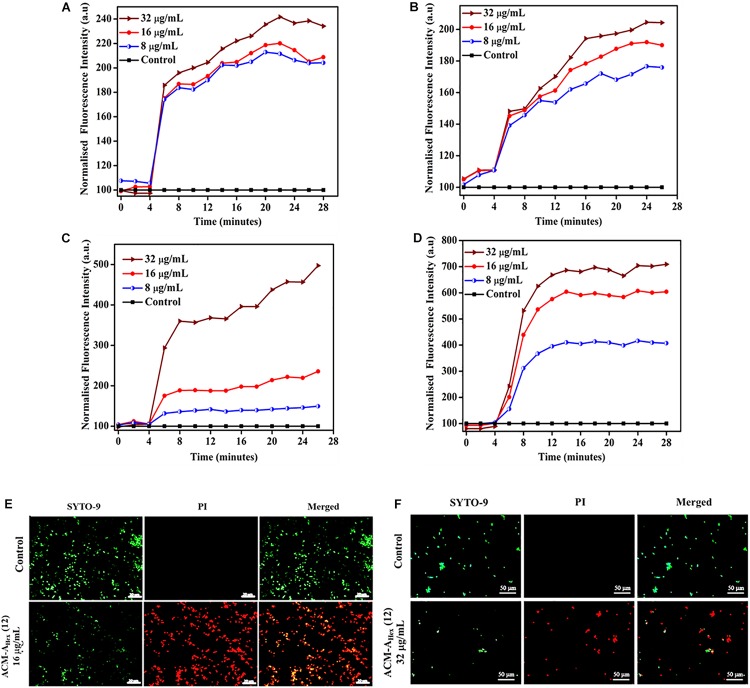
Membrane active mechanism of action by ACM-A_Hex_ (**12**)**:** membrane depolarization against **(A)** growing planktonic MRSA ATCC33591; **(B)** stationary phase MRSA ATCC33591; Membrane depolarization against **(C)** *C. albicans* ATCC10231 and **(D)** *C. albicans* AB226. **(E)** Life-Dead assay against MRSA ATCC33591. Scale bar is 20 μm. **(F)** Life-Dead assay against *C. albicans* ATCC10231. Scale bar is 50 μm.

As a preliminary mechanism of action, membrane depolarizing ability of the lead molecule was investigated against both bacteria (MRSA) and fungi (*C. albicans*) (Figure 7). In addition to the MRSA planktonic cells, membrane activity of the compound was also studied against metabolically inactive stationary phase cells. This study was performed using membrane-potential sensitive dye 3,3′-dipropylthiadicarbocyanine iodide [DiSC_3_ (**5**)]. This dye distributes both inside and outside microbial cell under normal potential across the membrane. Thus, the fluorescence intensity decreases owing to its self-quenching inside the microbial cells. Membrane active agent which can perturb the membrane potential leads to the release of such dye from interior to the exterior part of bacterial cells. This results in the enhancement of the fluorescence intensity gradually with time. Herein, addition of various concentrations of the lead compound ACM-A_Hex_ (**12**) (8, 16, and 32 μg/mL) resulted an increment in fluorescence intensity of DiSC_3_ (**5**) for bacteria (both planktonic and stationary phase cells) (Figures 7A,B) as well as for fungi (Figures 7C,D). The extent of fluorescence enhancement followed a concentration dependency (greater effect at higher concentration of the compound). These results establish that the macromolecule primarily target the microbial membrane for killing through perturbing the membrane polarization.

### Live/Dead Assay Against Bacteria and Fungi

In order to visualize the extent of bacterial and fungal killing qualitatively, we performed fluorescence microscopy using SYTO-9 and PI. These results exhibited the presence of dead microbial cells corresponding to red fluorescence upon treatment with lead compound ACM-A_Hex_ (**12**) at 16 and 32 μg/mL against bacteria and fungi respectively (Figures 7E,F). In case of the untreated control bright green fluorescence was observed indicating almost all the cells were alive.

### Development of Resistance Propensity

**FIGURE 8:**
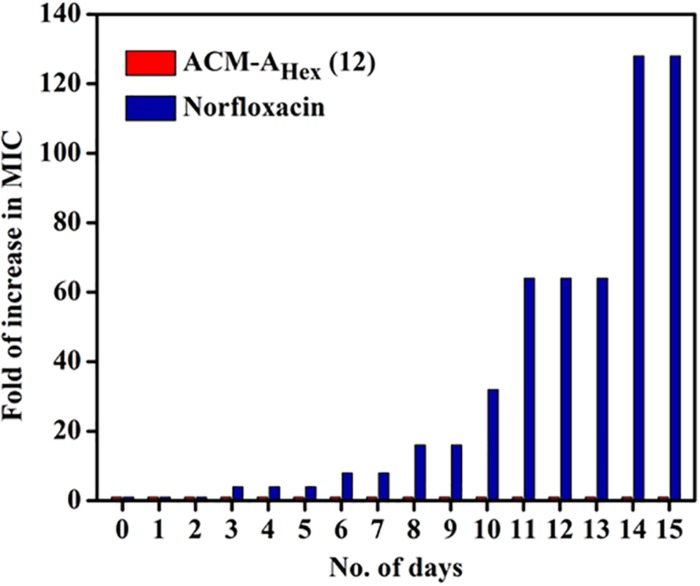
Propensity of resistance development of MRSA ATCC33591 against ACM-A_Hex_ (**12**) and Norfloxacin. In each passage, MIC was performed in triplicate and reproducible experimental results were obtained (*p* = 0.0147, Student’s *t*-test with Welch’s correction).

Resistance development in bacteria against the conventional antibiotics has become a serious concern worldwide. Hence, antibacterial agent with sustained activity is highly desirable in clinical settings. Toward this goal, propensity of resistance development in bacteria (MRSA ATCC33591) was studied against the lead compound, ACM-A_Hex_ (**12**) with respect to norfloxacin for 15 days. It was observed that bacteria could not develop any detectable resistance against the compound after continuous exposure for 15 days whereas the known antibiotic to treat MRSA infection norfloxacin displayed a 32-fold increment in MIC from 1 μg/mL after 8 days (Figure 8). After 15 days of serial passages bacteria developed a high level of resistance against norfloxacin with 128-fold increase in MIC. This result suggested that the compound is suitable for prolonged use in clinical settings.

## Discussion

Pathogenesis caused by drug-resistant Gram-positive bacteria such as MRSA, VRSA and VRE and their biofilm forming capability have created an alarming situation in the current scenario (Cantas et al., 2013; Brown and Wright, 2016; Cheng et al., 2016). Alongside, fungal infections majorly caused by *C. albicans* are also prevalent in terms of their severity to cause huge mortality and morbidity. In addition to these, polymicrobial infections owing to the co-existence of MRSA and *C. albicans* are another major threat in the face of global public health (Harriott and Noverr, 2009, 2011; Lohse et al., 2018). The situation has further been deteriorated by the evolutionary pressure of microbial resistance to the last resort antibiotics and anti-fungal drugs.

In this direction, numerous AMP mimicking membrane active agents has been developed by various groups all over the world. Most of the cases, the potency of such membrane active molecules was investigated against a broad spectra of pathogenic bacteria (Dhanda et al., 2019; Ghosh et al., 2019; Gupta V. K. et al., 2019). Antifungal efficacy was documented for very few amphiphilic polymers such as poly β-lactam and peptidopolysaccharides (Li et al., 2012; Liu et al., 2015). However, antimicrobial agent with dual efficacy to combat polymicrobial infections caused by MRSA and *C. albicans* has rarely been reported (Gupta S. et al., 2019). In this regard, we have designed a new class of amphiphilic cationic macromolecules (ACMs) based on branched polyethyleneimine (PEI) through a simple two-step post-functionalization strategy. In the molecular design, small molecular weight PEI (∼600 Da) has been used in order to obtain a less cytotoxic antimicrobial agent. Another rationale behind choosing such backbone with small molecular weight was to achieve final macromolecules (3.5–6 kDa) with molecular weight less than 50 kDa, a maximum threshold for renal clearance.

An optimum amphiphilic balance (hydrophobicity/hydrophilicity) is a crucial parameter for an antimicrobial agent with superior selectivity toward microbes over mammalian cells. In order to do that, various normal alkyl, alkyl esters, and alkyl amides functionalities has been incorporated in the molecular design. A thorough structure-activity relationship (SAR) was accomplished which suggested an increase of antibacterial activity with increasing hydrophobicity. In case of macromolecules bearing normal alkyl chain, moving from shorter hydrophobic butyl (ACM-N_But_; **4**) to hexyl (ACM-N_Hex_; **5**) to octyl (ACM-N_Oct_; **6**) chain antimicrobial activity was found to be increased gradually. Interestingly, the increment of hydrophobicity did not result any increment in the hemolytic activity (HC_50_) for these macromolecules. Although, the molecule consisting decyl chain, ACM-N_Dec_ (**7**) was moderately toxic toward hRBCs despite displaying a broad spectrum antimicrobial activity. In case of macromolecule bearing alkyl ester (ACM-E_Alk_; **8–10**) and amide (ACM-A_Alk_; **11–13**) functionalities, we also observed a similar trend of increasing antimicrobial activity and toxicity with increasing hydrophobicity. However, macromolecule with alkyl amide analogs showed improved activity-toxicity profile in comparison to the molecules with same alkyl ester moiety. This observation is possibly due to the additional hydrogen bonding interaction with the microbial membrane lipids which is originated from the amide functionality present in the macromolecular pendant chain (Uppu et al., 2016).

Afterward, suitability for wide range of application of this class of macromolecules motivated us to find out their therapeutic indices (SI = HC_50_/MIC) against pathogenic microbes over mammalian cells. Herein, antimicrobial activity of ACM-N_Oct_ (**6**), ACM-N_Dec_ (**7**), ACM-E_Hex_ (**9**) and ACM-A_Hex_ (**12**) can be considered as a good reflection of potentiality against MRSA and *C. albicans* in comparison to their toxic effect toward mammalian cells. Thus, a high therapeutic index was observed for these four compounds.

In a recent report, the world health organization (WHO) identified MRSA, VRSA, and VRE as a top high priority pathogen considering their severity to cause difficult-to-treat infections. Furthermore, drug-resistant *C. albicans* are also responsible for complicated invasive infections. Hence, drug-resistant clinical isolates of both Gram-positive bacteria and fungi were challenged with the aforementioned four best selective compounds. In this context, molecule consisting aliphatic decyl long chain ACM-N_Dec_ (**7**) and hexyl amide conjugated macromolecule ACM-A_Hex_ (**12**) displayed superior activity. In following studies, we found that hexyl amide bearing molecule, ACM-A_Hex_ exhibited relatively better cell viability of HEK-293 cells compared to the molecule devoid of amide functionality (ACM-N_Dec_). This result indicated that the incorporation of amide moieties in the macromolecular design increased the biocompatibility further. Hence, ACM-A_Hex_ was selected as the optimized lead molecule for further studies in detail.

The molecular structure of the lead compound revealed the presence of amide linkages which connects the *N*-methylated PEI backbone with the pendant alkyl chain. Thereby, we assumed that this molecule may not retain its activity due to non-specific interactions with the proteins and degradation by amidases present in complex physiological fluids such as human plasma and mice liver homogenate. Interestingly, pre-incubation for shorter time period with these complex fluids resulted almost no change in MIC. We believe that retention of MIC may be due to the presence of non-peptidic amide linkage in the molecular architecture, possibly not recognizable by the proteases present in the aforementioned fluids. Even pre-incubation for longer time period (24 h) exhibited marginally twofold increase of MIC. This may be resulted due to non-specific interactions with the various complex components (proteins, enzymes etc.) present in the physiological fluids.

It was also found that, the compound was rapidly bactericidal and fungicidal in nature at different therapeutic concentrations. This rapid killing indicated that the molecule possibly has membrane targeting mode of action. Noticeably, most challenging stationary cells (MRSA and VRE) and persister cells (*S. aureus*) were completely eliminated very fast by this compound at similar concentrations. The compound exhibited potent activity against both planktonic cells and metabolically inactive (stationary cells) and antibiotic tolerant cells (persister) due to the membrane active mode of actions caused by non-specific interactions of cationic lipophilic macromolecules with negatively charged bacterial cell envelope.

Additionally, this class of compound was also able to eradicate preformed rigid biofilm of MRSA. This indicated that the compound has enough membrane active nature so that it disrupts the extracellular matrix of biofilm which contains various negatively charged polysaccharides, lipids, nucleic acid components, peptides etc. It is well established that biofilm consists of different metabolically inactive bacterial population. As the compound can kill metabolically inactive bacteria therefore along with biofilm disruption, it was efficient enough to kill different bacterial population embedded within the biofilm. It was interesting to observe that this class of macromolecule not only kill planktonic and metabolically inactive cells embedded within biofilm but also killed dispersed cells originated from biofilm. Whereas, vancomycin could only inactivate planktonic bacteria and was incompetent in killing dispersed cells and bacteria within biofilm.

Over the emergence of individual Gram-positive bacterial and fungal burden, co-existence of MRSA and *C. albicans* is prevalent in majority of nosocomial infections associated with *C. albicans* (Harriott and Noverr, 2009, 2011; Lohse et al., 2018) This co-existence resulted the formation of polymicrobial biofilms for which treatment options are limited. A broad spectrum antimicrobial activity of the lead compound against a wide range of Gram-positive bacteria and fungi motivated us to evaluate its efficacy to eradicate such polymicrobial biofilm. The compound indeed exhibited a significant reduction of both MRSA and *C. albicans* embedded in the polymicrobial biofilm. Needless to mention that efficacy against multi-species biofilm is a significant contribution in the field.

The studies to investigate the membrane active modes of action revealed that our compound perturbed the membrane potential of both bacteria and fungi. PI straining of bacterial cells in presence of our compound further proved its membrane permeablizing ability. It was also able to permeablize the fungal cells, proved by substantial PI staining upon compound treatment. More importantly, bacteria did not develop any detectable resistance against these class amphiphilic cationic molecules possibly due to non-specific membrane active mode of action. Whereas, bacteria developed a high level of resistance toward known antibiotic norfloxacin within 2 weeks.

## Conclusion

In conclusion, we have developed a new class of water soluble polyetheleneimine based ACMs by involving minimal simple synthetic steps through post-functionalization strategy. In general, this new class of macromolecules displayed selective antimicrobial activity against both drug-resistant Gram-positive bacteria and fungi over the mammalian cells. The lead macromolecule (ACM-A_Hex_: **12**) displayed a broad spectrum antimicrobial activity while exhibiting no hemolysis at the active concentration. Noticeably, unlike the AMPs, it retained the antibacterial activity even after incubation with complex physiological fluids. Particularly, it was capable of killing most challenging metabolically inactive stationary phase cells and persisters of MRSA. It also demonstrated the ability to eradicate preformed rigid biofilms of MRSA along with their dispersed cells. In addition to these, one of the important highlights of this molecule is its potential to eliminate polymicrobial biofilms formed by a mixed population of *C. albicans* and MRSA. Interestingly, bacteria were not able to develop resistance against the lead molecule possibly due to its membrane targeting mode of action. As per the best of our knowledge, dual efficacy of this class of compound to tackle bacteria and fungi, proficiency to kill metabolically inactive bacteria, disruption of single and multi-species biofilm and elimination of distinct dispersed bacterial cell is a significant contribution in the field of antimicrobial research. Altogether, the overall results suggested that this class of membrane targeting macromolecules bears an immense potential to be developed as a promising future therapeutic to tackle infections associated with the co-existence of bacteria and fungi.

## Data Availability Statement

All datasets generated for this study are included in the article/Supplementary Material.

## Ethics Statement

All the animal studies were performed in agreement with the Guidelines for Care and Use of Laboratory Animals of Jawaharlal Neheru Center for Advanced Scientific Research (JNCASR) and permitted by the Animal Ethics Committee of JNCASR.

## Author Contributions

SM and JH designed the project. SM, SB, and RM performed the experiments and analyzed the data. SM, SB, and JH wrote the manuscript.

## Conflict of Interest

The authors declare that the research was conducted in the absence of any commercial or financial relationships that could be construed as a potential conflict of interest.
