# Supplementary material for: Amphiphilic Cationic Macromolecules Highly Effective Against Multi-Drug Resistant Gram-Positive Bacteria and Fungi With No Detectable Resistance
Source: Front Bioeng Biotechnol. 2020 Feb 14;8:55. doi: 10.3389/fbioe.2020.00055 (PMC7033416; doi:10.3389/fbioe.2020.00055)
Supplement: Supplementary file 1 [file Data_Sheet_1.pdf]

Supplementary Material

Characterizations of *N*-methylated PEI (1)

FT-IR (cm^{-1}): 2953 and 2833 (C-H str), 1458 (C-H bend), ^{1}H-NMR (400 MHz, $CDCl_3$): δ/ppm 2.25 (s, 3H, –N(CH_3)-), 2.43-2.6 (m, 4H, -N(CH_2CH_2)-).

Characterizations of activated esters and amides intermediates (2a-2c and 3a-3c)

Butyl 2-bromoethanoate (2a): Yield, 97%; ^{1}H-NMR (400 MHz, $CDCl_3$): δ/ppm 0.89 (t, 3H terminal –CH_3), 1.34 (m, 2H, -CH_2*CH_2*CH_3), 1.56 (m, 2H, - CH_2*CH_2*CH_2CH_3), 3.88 (s, 2H, -CO*CH_2*Br), 4.18 (t, 2H, -COO*CH_2*-).

Hexyl 2-bromoethanoate (2b): Yield, 98%; ^{1}H-NMR (400 MHz, $CDCl_3$): δ/ppm 0.88 (t, 3H, terminal –CH_3), 1.28 (m, 6H, -CH_2(CH_2)$_3$-), 1.61 (m, 2H, -*CH_2*(CH_2)$_3CH_3$), 3.89 (s, -CO*CH_2*Br, 2H), 4.14 (t, 2H, -COO*CH_2* -).

Octyl 2-bromoethanoate (2c): Yield, 95%; ^{1}H-NMR (400 MHz, $CDCl_3$): δ/ppm 0.89 (t, 3H, terminal –CH_3), 1.28 (m, 10H -CH_2(CH_2)$_5CH_3$), 1.62 (m, 2H, -*CH_2*(CH_2)$_5CH_3$), 3.92 (s, 2H, -CO*CH_2*Br), 4.20 (t, 2H, -COO*CH_2*-).

***N*-butyl-2-bromoethanamide (3a):** Yield, 100%; ^{1}H-NMR (400 MHz, $CDCl_3$): δ/ppm 0.88 (t, 3H, terminal –CH_3), 1.35 (m, 2H, -CH_2*CH_2*CH_3), 1.56 (m, 2H, -CH_2*CH_2*CH_2CH_3), 3.29 (q, 2H, -CONH*CH_2*-,), 3.92 (s, 2H, -CO*CH_2*Br), 6.72 (br. s, 1H, amide –*NH*CO-).

***N*-hexyl-2-bromoethanamide (3b):** Yield, 96%; ^{1}H-NMR (400 MHz, $CDCl_3$): δ/ppm 0.91 (t, terminal 3H, – CH_3), 1.3 (m, 6H, -CH_2(CH_2)$_3$-), 1.49 (m, 2H, -*CH_2*(CH_2)$_3CH_3$), 3.31 (q, 2H, -CONH*CH_2*-), 3.88 (s, 2H, - CO*CH_2*Br), 6.54 (br. s, 1H, amide –*NH*CO-).

***N*-octyl-2-bromoethanamide (3c) :** Yield, 95.5%; ^{1}H-NMR (400 MHz, $CDCl_3$): δ/ppm 0.88 (t, terminal 3H, -CH_3), 1.28 (m, 10H - CH_2(CH_2)$_5CH_3$), 1.61 (m, 2H, -*CH_2*(CH_2)$_5CH_3$), 3.29 (q, 2H, -COO*CH_2*-), 3.94 (s, 2H, -CO*CH_2*Br), 7.22 (br. s, 1H, amide –*NH*CO-).

Characterizations of the ACM-N_{alk} (4-7)

ACM-N$_{But}$ (4): Yield, 83.4%; ^{1}H NMR (400 MHz, D_2O): δ/ppm 0.96 (t, 3H, terminal -*CH_3*), 1.38-1.51 (br , 2H, -CH_2*CH_2*CH_3), 1.72-1.89 (br , 2H, -CH_2*CH_2*$CH_2N(CH_3)CH_2CH_2$-), 3.12-3.6 (br, 9H, -(*CH_2*)$_2$N(*CH_2*)(*CH_3*)-).

ACM-N$_{Hex}$ (5): Yield, 84%; ^{1}H NMR (400 MHz, D_2O): δ/ppm 0.85 (t, 3H, terminal -*CH_3*), 1.18-1.48 (br, 6H, -CH_2(*CH_2*)$_3$-), 1.70-1.85 (br, 2H, -*CH_2*$CH_2N(CH_3)CH_2CH_2$-), 3.41-4.09 (br, 9H, -(*CH_2*)$_2$N(*CH_2*)(*CH_3*)-).

ACM-N$_{Oct}$ (6): Yield, 82.5%; ^{1}H NMR (400 MHz, D_2O): δ/ppm 0.88 (t, 3H, terminal -*CH_3*), 1.16-1.50 (br, 10H, -CH_2(*CH_2*)$_{5-)}$, 1.59-1.89 (br, 2H, -*CH_2*$CH_2N(CH_3)CH_2CH_2$-), 3.17-4.44 (br, 9H, -(*CH_2*)$_2$N(*CH_2*)(*CH_3*)-).

ACM-N$_{Dec}$ (7): Yield, 80.3%; ^{1}H NMR (400 MHz, $CDCl_3$): δ/ppm 0.88 (t, 3H, terminal -*CH_3*), 1.22-1.48 (br, 14H, -CH_2(*CH_2*)$_{7-}$), 1.6-1.75 (br, 2H, -*CH_2*$CH_2N(CH_3)CH_2CH_2$)-), 3.3-4.04 (br, 9H, -(*CH_2*)$_2$N(*CH_2*)(*CH_3*)-).

Characterizations of the ACM-E$_{alk}$ (8-10) and ACM-A$_{alk}$ (11-13)

ACM-E$_{But}$ (8): Yield, 76.5%; ^{1}H NMR (400 MHz, D_2O): δ/ppm 0.88 (t, 3H, terminal -*CH_3*), 1.22-1.35 (br, 2H, -CH_2*CH_2*CH_3), 1.65-1.75 (q, 2H, -CH_2*CH_2*$CH_2N(CH_3)CH_2CH_2$-), 3.52-3.85 (br, 7H, -(*CH_2*)$_2$N(CH_2)(*CH_3*)-), 3.9-4.4 (br, 2H, -*CH_2*$OCOCH_2N(CH_3)CH_2CH_2$-), 4.5-4.75 (br, 2H,- OCO*CH_2* $N(CH_3)CH_2CH_2$-).

ACM-E$_{Hex}$ (9): Yield, 76%; ^{1}H-NMR (400 MHz, $CDCl_3$): δ/ppm 0.89 (t, 3H, terminal -*CH_3*), 1.24-1.39 (br m, 6H, -CH_2(*CH_2*)$_3$-, 1.7-1.85 (q, 2H, - *CH_2*$CH_2N(CH_3)CH_2CH_2$-), 3.79-4.29 (m, 7H, -(*CH_2*)$_2$N(CH_2)(*CH_3*)-), 4.35-4.6 (m, 2H, -*CH_2*$OCOCH_2N(CH_3)CH_2CH_2$-), 4.55-4.74 (m, 2H, -OCO*CH_2* $N(CH_3)CH_2CH_2$-).

ACM-E$_{Oct}$ (10): Yield, 75.4%; ^{1}H NMR (400 MHz, $CDCl_3$): δ/ppm 0.87 (t, 3H, terminal -*CH_3*), 1.28-1.38 (br, 10H, -CH_2(*CH_2*)$_5$-), 1.75-1.87 (q, 2H, -*CH_2*$CH_2N(CH_3)CH_2CH_2$-), 3.6-3.8 (br, 7H, -(*CH_2*)$_2$N(CH_2)(*CH_3*)-), 4.1-4.45 (br, 2H, -*CH_2*$OCOCH_2N(CH_3)CH_2CH_2$-), 4.55-4.89 (br, 2H, -OCO*CH_2* $N(CH_3)CH_2CH_2$-).

ACM-A$_{But}$ (11): Yield, 76.5%; ^{1}H NMR (400 MHz, D_2O): δ/ppm 0.85 (t, 3H, terminal -*CH_3*), 1.28-1.48 (br, 2H,- CH_2*CH_2*CH_3), 1.82 (q, 2H, -CH_2*CH_2*$CH_2N(CH_3)CH_2CH_2$-), 3.1-3.8 (br, 7H, -

($\mathit{CH_2}$)$_2$N(CH_2)($\mathit{CH_3}$)-), 3.9-4.4 (br, 2H, -$\mathit{CH_2}$NHCOCH_2N(CH_3)CH_2CH_2-), 4.5-4.9 (br, 2H, -NHCO$\mathit{CH_2}$ N(CH_3)CH_2CH_2-), 8.0-8.5 (br, 1H, -CO*NH*CH_2-).

ACM-A$_{\mathbf{Hex}}$ (12): Yield, 79%; ^{1}H NMR (400 MHz, $CDCl_3$): δ/ppm 0.88 (t, 3H, terminal -$\mathit{CH_3}$), 1.2-1.39 (br, 6H, -CH_2($\mathit{CH_2}$)$_3$-), 1.72- 1.9 (q, 2H, -$CH_2$$\mathit{CH_2}$$CH_2$N($CH_3$)$CH_2CH_2$-), 3.2-3.89 (br, 7H, -($\mathit{CH_2}$)$_2$N($CH_2$)($\mathit{CH_3}$)-), 4.05-4.3 (br, 2H, - $\mathit{CH_2}$NHCOCH_2N(CH_3)CH_2CH_2-), 4.5-4.86 (br, 2H,-NHCO$\mathit{CH_2}$ N(CH_3)CH_2CH_2-), 8.05-8.5 (br, 1H, -CO*NH*CH_2-).

ACM-A$_{\mathbf{Oct}}$ (**13**): Yield, 75.2%; ^{1}H NMR (400 MHz, $CDCl_3$): δ/ppm 0.89 (t, 3H, terminal -$\mathit{CH_3}$), 1.2-1.49 (br m, 10H, -CH_2($\mathit{CH_2}$)$_5$), 1.7-1.87 (q, 2H, -$CH_2$$\mathit{CH_2}$$CH_2$N($CH_3$)$CH_2CH_2$-), 3.1-3.67 (br, 7H, -($\mathit{CH_2}$)$_2$N($CH_2$)($\mathit{CH_3}$)-), 3.93.-4.3 (br, 2H, - $\mathit{CH_2}$NHCOCH_2N(CH_3)CH_2CH_2-), 4.6-4.8 (br, 2H, -NHCO$\mathit{CH_2}$ N(CH_3)CH_2CH_2-), 8-8.5 (br, 1H, -CO*NH*CH_2-).

Table S1.Molecular weight of the amphiphilic cationic macromolecules (ACMs).

**Macromolecules**	**[a]Degree of quaternization (DQ) (%)**	**[b]Molecular Weight (M_n) (kDa)**
**ACM-N_{but} (4)**	**100**	**3.6**
**ACM-N_{Hex} (5)**	**100**	**4.2**
**ACM-N_{Oct} (6)**	**100**	**4.7**
**ACM-N_{Dec} (7)**	**100**	**5.2**
**ACM-E_{But} (8)**	**100**	**4.7**
**ACM-E_{Hex} (9)**	**100**	**5.2**
**ACM-E_{Oct} (10)**	**100**	**5.7**
**ACM-A_{But} (11)**	**100**	**4.7**
**ACM-A_{Hex} (12)**	**100**	**5.2**
**ACM-A_{Oct} (13)**	**100**	**5.7**

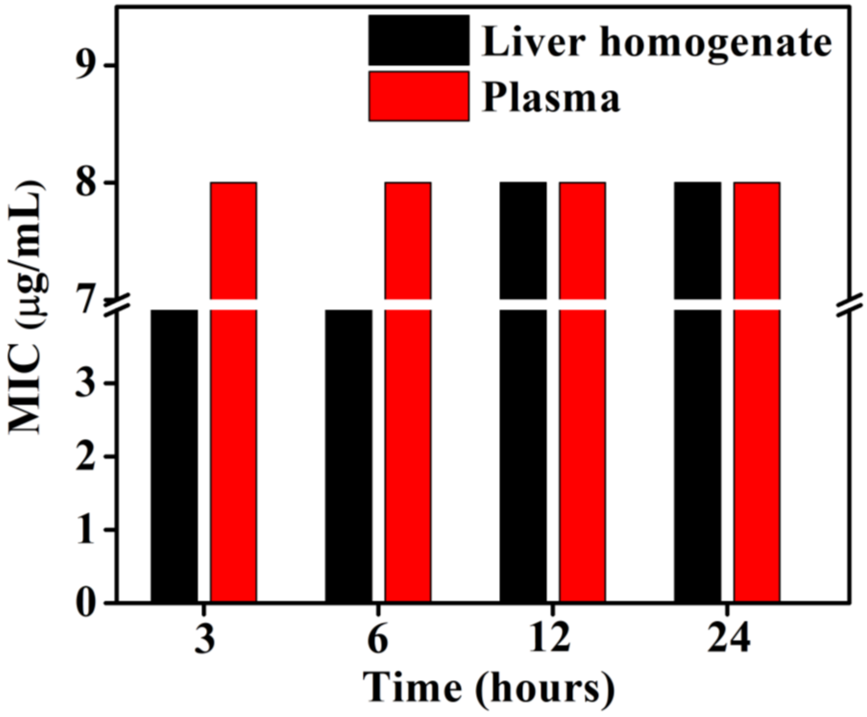

Figure S1. Antibacterial activity of ACM-A_{Hex} (**12**) in physiological fluids. Each concentration had triplicate values and the entire experiment was performed twice. The average data was reported.
